# Development of a quadruplex real-time quantitative RT-PCR for detection and differentiation of PHEV, PRV, CSFV, and JEV

**DOI:** 10.3389/fvets.2023.1276505

**Published:** 2023-10-31

**Authors:** Xin Hu, Shuping Feng, Kaichuang Shi, Yuwen Shi, Yanwen Yin, Feng Long, Xiankai Wei, Zongqiang Li

**Affiliations:** ^1^College of Animal Science and Technology, Guangxi University, Nanning, China; ^2^Guangxi Center for Animal Disease Control and Prevention, Nanning, China

**Keywords:** porcine hemagglutinating encephalomyelitis virus (PHEV), porcine pseudorabies virus (PRV), classical swine fever virus (CSFV), Japanese encephalitis virus (JEV), multiplex qRT-PCR

## Abstract

Porcine hemagglutinating encephalomyelitis virus (PHEV), porcine pseudorabies virus (PRV), classical swine fever virus (CSFV), and Japanese encephalitis virus (JEV) cause similar neurological symptoms in the infected pigs, and their differential diagnosis depends on laboratory testing. Four pairs of specific primers and probes were designed targeting the PHEV N gene, PRV gB gene, CSFV 5′ untranslated region (5’UTR), and JEV NS1 gene, respectively, and a quadruplex real-time quantitative RT-PCR (qRT-PCR) was developed to detect and differentiate PHEV, PRV, CSFV, and JEV. The assay showed high sensitivity, with the limit of detection (LOD) of 1.5 × 10^1^ copies/μL for each pathogen. The assay specifically detected only PHEV, PRV, CSFV, and JEV, without cross-reaction with other swine viruses. The coefficients of variation (CVs) of the intra-assay and the inter-assay were less than 1.84%, with great repeatability. A total of 1,977 clinical samples, including tissue samples, and whole blood samples collected from Guangxi province in China, were tested by the developed quadruplex qRT-PCR, and the positivity rates of PHEV, PRV, CSFV, and JEV were 1.57% (31/1,977), 0.35% (7/1,977), 1.06% (21/1,977), and 0.10% (2/1,977), respectively. These 1,977 samples were also tested by the previously reported qRT-PCR assays, and the coincidence rates of these methods were more than 99.90%. The developed assay is demonstrated to be rapid, sensitive, and accurate for detection and differentiation of PHEV, PRV, CSFV, and JEV.

## 1. Introduction

Porcine hemagglutinating encephalomyelitis virus (PHEV), porcine pseudorabies virus (PRV), classical swine fever virus (CSFV), and Japanese encephalitis virus (JEV), which cause neurological symptoms in the infected pigs, are the very important pathogens that cause huge economic losses to Chinese swine industry each year.

Porcine hemagglutinating encephalomyelitis (PHE) was first reported in Ontario, Canada in 1957 (1). The etiological agent PHEV, one member of the genus *Betacoronavirus* in the family *Coronaviridae*, is an enveloped, single-stranded, positive-sense RNA virus (2). PHEV was first isolated from suckling piglets that suffered from encephalomyelitis in 1962 (3). PHEV can infect the pigs of all age, but the clinical symptoms and the degree of harms are related to age. Clinically, it is mainly divided into two types of neurological signs: vomiting and central nervous system dysfunction (encephalomyelitis), which mainly cause serious injury to under 4-week-old piglets, and the morbidity rate and the mortality rate are age-dependent, and the fatality rate is as high as 100% (4). According to serological investigation, PHEV is still prevalent among pig herds in many countries in the world, but usually shows subclinical infections (5, 6). In China, PHEV was first reported in 1986, and has been discovered in several provinces since then (7, 8).

PRV, an enveloped, double-stranded DNA virus, belongs to the genus *varicellovirus* in the family *Herpesviridae* (9). PRV can infect a variety of mammals, including ruminants, rodents, and predators, of which pig acts as the viral natural host and potential carrier. PRV causes pseudorabies (PR) in the infected pigs, usually showing respiratory symptoms in the adult pigs, and central nervous system diseases in the piglets (9). PR was first described in 1813 in America, and has been prevalent in pigs around the world for more than 200 years (10, 11). Nowadays, vaccination with the gE-gene-deleted vaccine is the most effective measure to prevent and control this disease and minimize its economic losses (12, 13). However, PR has not been completely eradicated in many countries since lots of PRV variant strains have been appearing constantly in recent years (13, 14).

CSFV, one member of the genus *Pestivirus* in the family *Flaviviridae*, is an enveloped, single-stranded, positive sense RNA virus (15). Classical swine fever (CSF) was first reported in Tennessee in the United States of American (USA) in 1810 (16). CSF is a highly contagious infectious disease, which is characterized with high fever, anorexia, drowsiness, and respiratory, digestive tract and nervous system symptoms. The CSFV infected pregnant sows might show abortion, production of mummified fetuses, stillbirth, and deformed fetuses (17). Currently, CSF has been steadily controlled by administering different CSFV vaccines worldwide, and CSF has been eradicated in Australia, Canada, USA, and many European countries (18, 19). However, CSF is still sporadic or prevalent in many countries in Asia, South America, Africa and Europe, and cause huge economic losses in these countries every year (20, 21).

JEV, one member of the genus *Orthoflaviviru*s in the family *Flaviviridae*, is an enveloped, single-stranded, positive sense RNA virus (22). JEV is an important zoonotic etiology which causes severe acute encephalitis and nervous system syndromes in humans and animals (23). It was first reported in 1871 in Japan (24). Pig is the main amplifying host of JEV, and Culex tritaeniorhynchus is the vector to transmit this pathogen (25). Pigs infected with JEV generally have no obvious symptoms, but there are also reports of fever, anorexia, and reproductive disorders (26). At present, the most effective measure to prevent and control JE in pigs is to vaccinate with effective vaccines (27), but JE still occurs occasionally in some pig herds (28, 29).

Since PHEV, PRV, CSFV, and JEV cause similar clinical neurological symptoms, and there might have co-infection and/or secondary infection of these pathogens, it is hard to distinguish and diagnose these diseases only on the basis of the clinical manifestations and pathological damages. So, specific, accurate, and reliable detection and differentiation of these viruses is necessary to accurately diagnose these diseases, and take timely and effective prevention and control measures. The real-time quantitative PCR/RT-PCR (qPCR/qRT-PCR) has the advantages of high sensitivity, excellent specificity, high throughput, uneasy contamination, and convenient operation, and is widely used to detect viral nucleic acids (30). To date, the qPCR/qRT-PCR has been reported to detect PHEV (31), PRV (32, 33), CSFV (34, 35), and JEV (36, 37), and the multiplex qRT-PCR has been reported to detect PHEV, PRV, CSFV, and/or JEV (38–40). However, no multiplex qRT-PCR to simultaneously detect and differentiate PHEV, PRV, CSFV, and JEV has been reported until now. Since the multiplex qPCR can detect multiple targets at one reaction, so can greatly save time and effort, and is the preferred method for laboratories to simultaneously detect multiple viral nucleic acids. Therefore, the PHEV N gene, PRV gB gene, CSFV 5′ untranslated region (5'UTR), and JEV NS1 gene were used as the targets for design of the specific primers and probes, and a quadruplex qRT-PCR was established for rapid, sensitive, and accurate detection of PHEV, PRV, CSFV, and JEV in this study.

## 2. Materials and methods

### 2.1. Vaccine viruses and positive samples

The vaccine strains of CSFV (C strain), PRV (Bartha-K61 strain), JEV (SA14-14-2 strain), porcine epidemic diarrhea virus (PEDV and CV777 strain), transmissible gastroenteritis virus (TGEV and H strain), porcine rotavirus (PoRV and NX strain), porcine reproductive and respiratory syndrome virus (PRRSV and CH-1R strain), porcine circovirus type 2 (PCV2 and WH strain), foot-and-mouth disease virus (FMDV and O/Mya98/XJ/2010 strain), and swine influenza virus (SIV and TJ strain) were purchased from Huapai Biological Group (Chengdu, China). The vaccine strains of CSFV (WH-09 strain and CVCC AV1412 strain), and PRV (HB-2000 strain, HN1201strain, and EA strain) were purchased from Wuhan Keqian Biology Corporation Limited (Wuhan, China).

The clinical positive samples of PHEV, porcine deltacoronavirus (PDCoV), African swine fever virus (ASFV), PCV3, Senecavirus A (SVA), and atypical porcine pestivirus (APPV), which were confirmed by PCR/RT-PCR and gene sequencing, were provided by our laboratory. The vaccine strains and the positive samples were stored at −80°C until used.

### 2.2. The clinical samples

From January 2021 to February 2023, a total of 1,977 clinical samples, including brain, liver, spleen, lung, and kidney from each pig (the tissue homogenate from each pig was considered as one sample when tested by the developed quadruplex qRT-PCR), were collected from 1,977 pigs from different pig farms, slaughterhouses, and death-pig harmless treatment plants in Guangxi province, southern China. The 1,828 tissue samples were collected from abnormal dead pigs in pig farms and harmless treatment plants, and the 149 whole blood samples were collected from pigs with depression, weight loss, anorexia, diarrhea, elevated body temperature, and/or neurological abnormalities/symptoms in pig farms and slaughterhouses. The samples were transported to our laboratory under ≤4°C within 8 h, and stored at −80°C until used.

### 2.3. Design of primers and probes

Four pairs of primers and probes (Table 1), which based on the sequences of PHEV (GenBank accession number: FJ009234.1), PRV (KU552118.1), CSFV (MT799518.1), and JEV (MN544780.1) published in GenBank of the NCBI,^1^ were designed to amplify PHEV N gene, PRV gB gene, CSFV 5′ untranslated region (5'UTR), and JEV NS1 gene, respectively. The multiple nucleotide alignments of the amplified targeted regions of these viruses are shown in Supplementary Figure S1. The targeted fragments of different viruses are shown in Supplementary Table S1.

**Table 1:** The specific primers and probes.

Name	Sequence (5′ → 3′)	Tm/°C	Product/bp
PHEV(N)-F	CCAGAAGGATGTTTATGAATTGC	54.1	119
PHEV(N)-R	CCTGATGTTGATAGGCATTCA	54.2	
PHEV(N)-P	FAM-TGGCGCGATTAGATTTGAYAGCACACTC-BHQ1	67.4	
PRV(gB)-F	ACGACAACGAGCTCCTCATCT	62.0	142
PRV(gB)-R	CTGATCGTCTCGGGCACCT	61.1	
PRV(gB)-P	VIC-TCATCGAGCCCTGCACCGGCAACCA-BHQ1	69.9	
CSFV(5'U)-F	GAGGGACTAGCCGTRGTGG	59.0	113
CSFV(5'U)-R	CCTCGTCCACRTAGCATCTCG	58.9	
CSFV(5'U)-P	CY5-AGCTCCCTGGGTGGTCTAAGTCCTGAGT-BHQ2	68.9	
JEV(NS1)-F	TTTCTGGCCACCCAGGAGG	60.8	101
JEV(NS1)-R	GTGATGCCCCCAAGCATCAG	61.6	
JEV(NS1)-P	ROX-CCTTCGCAAGAGGTGGACGGCCAGA-BHQ2	71.5	

### 2.4. Extraction of viral DNA/RNA

The vaccine viruses and the tissue homogenates which were resuspended in phosphate-buffered saline (PBS, pH 7.2) (20%, W/V), and the whole blood samples were vortexed at room temperature (5 min), and centrifuged at 4°C (12,000 rpm, 5 min). Two hundred microliters of the supernatants were used to extract the total viral DNA and RNA by the Viral RNA/DNA Extraction Kit Ver.4.0 (Tianlong, Xian, China), and the extracted nucleic acids were stored at −80°C until used.

### 2.5. Preparation of the standard plasmids

The viral DNA was extracted from the PRV vaccine strain, and the viral RNA were extracted from the PHEV positive sample, CSFV vaccine strain, and JEV vaccine strain, as described in Section 2.4. The viral RNAs were then reverse transcribed into cDNA using the Primescript II 1st Strand cDNA Synthesis Kit (TaKaRa, Dalian, China). The DNA/cDNA were used as templates to amplify the targeted fragments by PCR using the specific primers (Table 1). The PCR products were purified, and used to construct the standard plasmids as described by Chen et al. (41). The recombinant standard plasmids were confirmed by sequencing, named p-PHEV, p-PRV, p-CSFV, and p-JEV, respectively, and stored at −80°C until used.

The standard plasmids were quantified by a NanoDrop spectrophotometer (Thermo Fisher, Waltham, MA, United States) using the following formula: plasmid (copies/μL) = $\frac{\left(6.02\times 10^{23}\right)\times \left(X\;\mathrm{ng}/\mathrm{\mu L}\times 10^{-9}\right)}{\mathrm{plasmid}\;\mathrm{length}\;\left(\mathrm{bp}\right)\times 660}$ .

### 2.6. Optimization of the reaction conditions

The multiplex qRT-PCR was conducted in a 20 μL reaction volume. The following reagents from TaKaRa Biotechnology (Dalian) Co., Ltd. (Dalian, China) were used: 10 μL 2 × One-Step qRT-PCR Buffer III, 0.4 μL Ex Taq HS (5 U/μL), 0.4 μL PrimeScript RT Enzyme Mix II. In addition, 0.2 ~ 0.8 μL of the mixture of the primers and probes, 2.0 μL of the mixture of the four standard plasmids, and distilled water to a total volume of 20 μL were used. The optimization of the reaction conditions was carried out using the ABI QuantStudio™ 5 Real-Time System (ABI, Carlsbad, CA, United States) with the following steps: 42°C for 5 min, 95°C for 10 s, 40 cycles at 95°C for 5 s, 56°C-61°C for 30 s. The fluorescent signals were collected each cycle, and the maximum ∆Rn and the minimal cycle (Ct) values were obtained after the amplification.

### 2.7. Analysis of the standard curves

After mixing the four standard plasmids in equal volume, they were 10-fold serially diluted from 1.5 × 10^8^ to 1.5 × 10^2^ copies/μL (final concentration in the reaction system: 1.5 × 10^7^ to 1.5 × 10^1^ copies/μL), then used to make the standard curves of the developed assay.

### 2.8. Evaluation of the analytical specificity

The viral DNA/RNA of PEDV, TGEV, PoRV, PDCoV, ASFV, PRRSV, SIV, PCV2, PCV3, FMDV, SVA, and APPV were used as templates, the mixture of the standard plasmids p-PHEV, p-PRV, p-CSFV, and p-JEV, the negative tissue homogenate, and the nuclease-free distilled water were used as controls for evaluating the specificity of the developed assay.

### 2.9. Evaluation of the analytical sensitivity

After mixing the four standard plasmids in equal volume, they were 10-fold serially diluted from 1.5 × 10^8^ to 1.5 × 10^0^ copies/μL (final concentration in the reaction system: 1.5 × 10^7^ to 1.5 × 10^−1^ copies/μL), then used as templates to evaluate the sensitivity of the developed assay.

### 2.10. Evaluation of the repeatability

The coefficient of variation (CV) value was used for evaluation of the repeatability of the established assay. The mixtures of the four standard plasmids of 1.5 × 10^8^, 1.5 × 10^6^, 1.5 × 10^4^ copies/μL (final concentration in the reaction system: 1.5 × 10^7^, 1.5 × 10^5^, 1.5 × 10^3^ copies/μL) were used as templates. The CV value was calculated by performing the quadruplex qRT-PCR in triplicate for the intra-assay variability, and on three different days for the inter-assay variability.

### 2.11. Detection of the clinical samples

In order to evaluate the application of the developed assay for detection of the clinical samples, the total viral DNA/RNA were extracted from the 1,977 clinical tissue samples and whole blood samples from Guangxi province using the Viral RNA/DNA Extraction Kit Ver.4.0 (Tianlong, Xian, China), and used as templates to detect PHEV, PRV, CSFV, and JEV by the quadruplex qRT-PCR assay.

In addition, the 1,977 samples were also tested by the multiplex qRT-PCR developed by Wang et al. for detection of PHEV (31), and Wu et al. for detection of PRV, CSFV, and JEV (38). The positivity rates of PHEV, PRV, CSFV, and JEV by the developed assay in this study were compared with those of these two methods, and their coincidence rates were evaluated.

Furthermore, the samples were also tested by the qPCR (42) described in the WOAH’s Terrestrial Manual 2018 for PRV,^2^ the qRT-PCR (43) described in the WOAH’s Terrestrial Manual 2022 for CSFV,^3^ the RT-PCR (44) described in the WOAH’s Terrestrial Manual 2021 for JEV,^4^ and the qRT-PCR described in the local standards of Jilin province in China for PHEV^5^ in order to validate the application of the developed assay in this study.

## 3. Results

### 3.1. Preparation of the standard plasmids

The PCR products of the PHEV N gene, PRV gB gene, CSFV 5'UTR, and JEV NS1 gene were purified, and used to construct the standard plasmids. After confirming by sequencing, these plasmids were named p-PHEV, p-PRV, p-CSFV, and p-JEV, respectively. The sequences of the amplified targeted fragments are shown in Supplementary Table S1. Their concentrations were determined to be 1.51 × 10^10^, 5.18 × 10^10^, 8.91 × 10^10^, and 4.50 × 10^10^ copies/μL, respectively. They were diluted to 1.50 × 10^10^ copies/μL, and stored at −80°C until used.

### 3.2. Determination of the optimal parameters

The reaction system is composed of primers, probes, enzymes, and nuclease-free distilled water. After optimizing the reaction conditions, the optimal parameters of the quadruplex qRT-PCR were obtained (Table 2). The amplification procedures were as follows: 42°C for 5 min, 95°C for 10 s, and 40 cycles of 95°C for 5 s and 60°C for 30 s. The sample with a Ct value ≤35 cycles was judged as positive, while with a Ct value >35 cycles was judged as negative.

**Table 2:** The components and the optimal parameters.

Regent	Volume (μL)	Final concentration (nM)
2× One-Step RT-PCR Buffer III	10	/
Ex Taq HS (5 U/μL)	0.4	/
PrimerScript RT Enzyme Mix II	0.4	/
PHEV(N)-F (20 pmol/μL)	0.2	200
PHEV(N)-R (20 pmol/μL)	0.2	200
PHEV(N)-P (20 pmol/μL)	0.3	300
PRV(gB)-F (20 pmol/μL)	0.3	300
PRV(gB)-R (20 pmol/μL)	0.3	300
PRV(gB)-P (20 pmol/μL)	0.3	300
CSFV(5'U)-F (20 pmol/μL)	0.5	500
CSFV(5'U)-R (20 pmol/μL)	0.5	500
CSFV(5'U)-P (20 pmol/μL)	0.4	400
JEV(NS1)-F (20 pmol/μL)	0.2	200
JEV(NS1)-R (20 pmol/μL)	0.2	200
JEV(NS1)-P (20 pmol/μL)	0.2	200
Total Nucleic Acids	2.0	/
RNase-Free Distilled H_2_O	Up to 20	/

### 3.3. Generation of the standard curves

**Figure 1:**
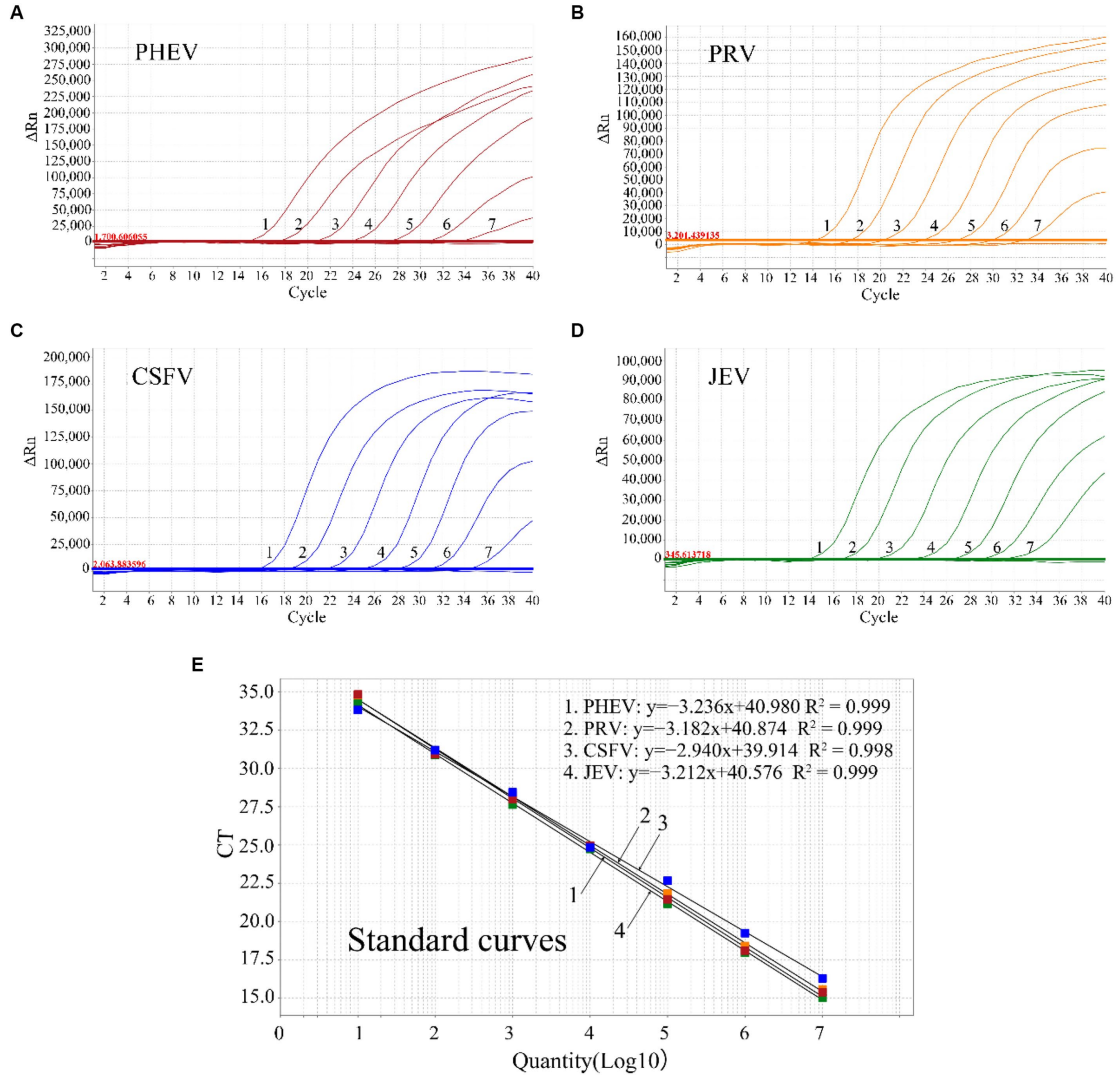
The amplification curves of p-PHEV **(A)**, p-PRV **(B)**, p-CSFV **(C)**, and p-JEV **(D)** with final reaction concentration from 1.5 × 10^7^ to 1.5 × 10^1^ copies/μL (1–7), and the standard curves of the quadruplex qRT-PCR **(E)**.

The mixture of the four plasmids from 1.5 × 10^8^ to 1.5 × 10^2^ copies/μL (final concentration in the reaction system: 1.5 × 10^7^ to 1.5 × 10^1^ copies/μL) was used to generate the standard curves of the quadruplex assay. The results indicated that the slope, *R*^2^, and Eff% were − 3.236, 0.999, 103.72% for PHEV, −3.182, 0.999, 106.195% for PRV, −2.94, 0.998, 118.827% for CSFV, and −3.212, 0.999, 104.801% for JEV, respectively (Figure 1), indicating a high correlation between Ct values and template concentrations.

### 3.4. Specificity

**Figure 2:**
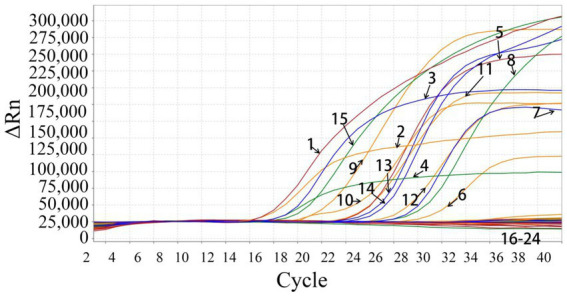
Specificity evaluation of the quadruplex qRT-PCR. 1: p-PHEV; 2: p-PRV; 3: p-CSFV; 4: p-JEV; 5: PHEV (clinical positive sample); 6: PRV (clinical positive sample); 7: CSFV (clinical positive sample); 8: JEV (clinical positive sample); 9: PRV Bartha-K61 strain; 10: PRV HB-2000 strain; 11: PRV HN1201 strain; 12: PRV EA strain; 13: CSFV CVCC AV1412 strain; 14: CSFV WH-09 strain; 15: JEV SA14-14-2 strain; 16–22: PEDV CV777 strain, TGEV H strain, PoRV NX strain, PRRSV CH-1R strain, PCV2 WH strain, FMDV O/Mya98/XJ/2010 strain, SIV TJ strain; 23: Clinical negative tissue sample; 24: Negative control (nuclease-free distilled water).

The swine viruses, PEDV, TGEV, PoRV, PDCoV, ASFV, PRRSV, SIV, PCV2, PCV3, FMDV, SVA, and APPV, were used to analyze the assay’s specificity. The developed assay generated specific amplification curves only from PHEV, PRV, CSFV, and JEV, and no fluorescent signal was obtained from the other 12 viruses, showing high specificity of the assay (Figure 2).

### 3.5. Sensitivity

**Figure 3:**
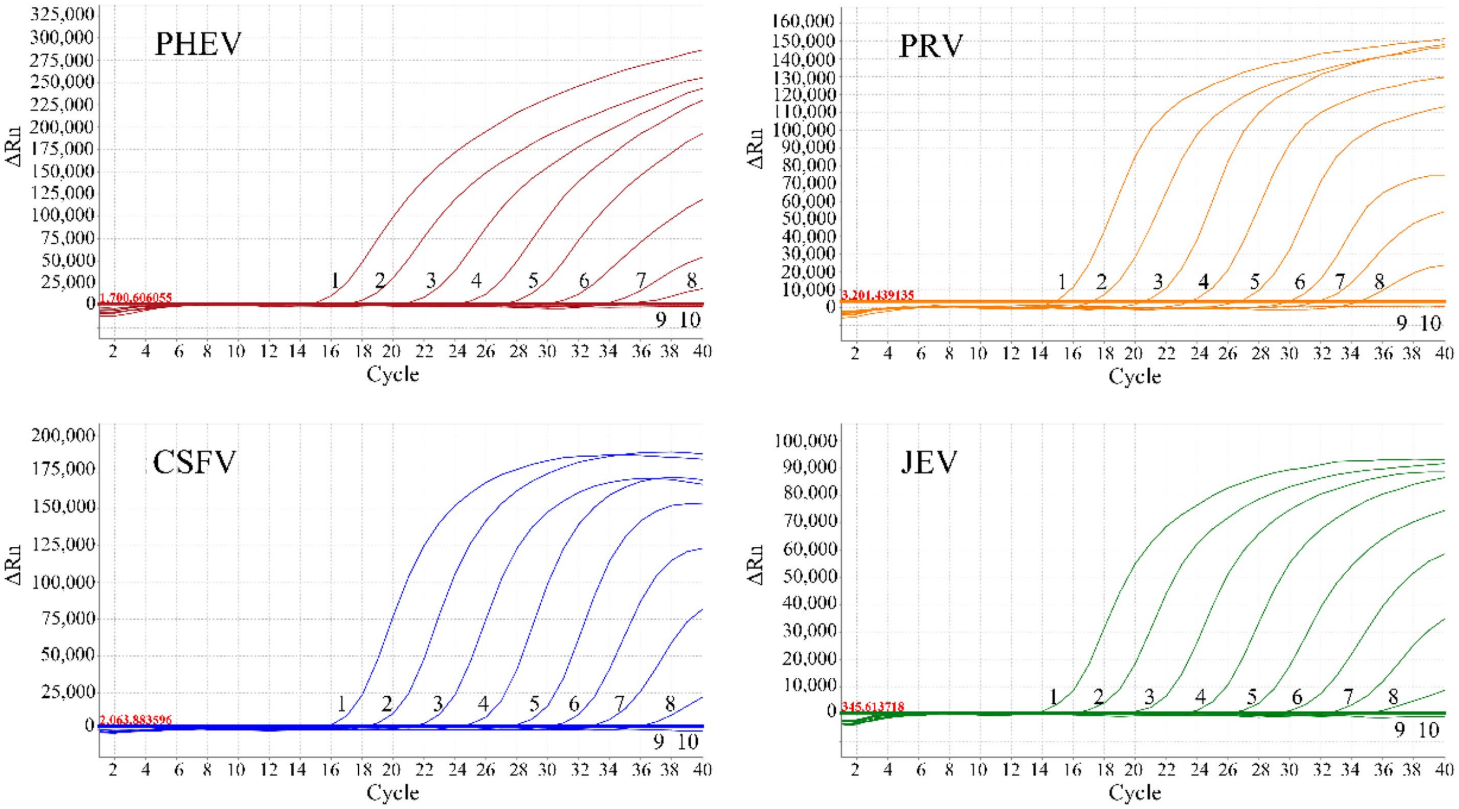
Sensitivity evaluation of the quadruplex qRT-PCR. 1–9: The amplification curves of each standard plasmid with final reaction concentrations from 1.5 × 10^7^ to 1.5 × 10^−1^ copies/μL; 10: Negative control.

The mixtures of the four plasmids with final reaction concentrations from 1.5 × 10^7^ to 1.5 × 10^−1^ copies/μL were used to evaluate the assay’s sensitivity. The results indicated that the limit of detection (LOD) of all the four plasmids were 1.5 × 10^1^ copies/μL (final reaction concentration), highlighting the high sensitivity of the quadruplex qRT-PCR (Figure 3).

### 3.6. Repeatability

Three gradient concentrations of p-PHEV, p-PRV, p-CSFV, and p-JEV (final reaction concentration: 1.5 × 10^7^, 1.5 × 10^5^, and 1.5 × 10^3^ copies/μL) were used to evaluate the assay’s repeatability. The coefficients of variation (CVs) of the intra-, and the inter-assay variability were less than 1.02, and 1.84%, respectively, indicating high repeatability of the assay (Table 3).

**Table 3:** Evaluation of the assay’s repeatability.

Plasmid	Concentration (Copies/μL)	Intra-assay			Inter-assay		
		$\bar{X}$	SD	CV (%)	$\bar{X}$	SD	CV (%)
p-PHEV	1.50 × 10^7^	14.65	0.10	0.65	14.26	0.05	0.35
	1.50 × 10^5^	20.30	0.10	0.50	20.03	0.21	1.04
	1.50 × 10^3^	26.85	0.01	0.02	27.39	0.10	0.38
p-PRV	1.50 × 10^7^	14.34	0.04	0.28	13.60	0.98	0.72
	1.50 × 10^5^	20.45	0.09	0.42	19.86	0.26	1.29
	1.50 × 10^3^	26.80	0.01	0.04	26.81	0.07	0.27
p-CSFV	1.50 × 10^7^	14.75	0.12	0.83	13.89	0.08	0.61
	1.50 × 10^5^	20.48	0.07	0.36	20.45	0.25	1.23
	1.50 × 10^3^	26.70	0.27	1.02	27.53	0.02	0.09
p-JEV	1.50 × 10^7^	14.19	0.06	0.43	13.82	0.19	1.38
	1.50 × 10^5^	20.47	0.08	0.40	20.50	0.38	1.84
	1.50 × 10^3^	26.94	0.05	0.20	27.45	0.21	0.77

### 3.7. Detection results of the clinical samples

The developed quadruplex qRT-PCR was used to detect the 1,977 clinical samples collected from Guangxi province of China, and the positivity rates of PHEV, PRV, CSFV, and JEV were 1.57% (31/1,977), 0.35% (7/1,977), 1.06% (21/1,977), and 0.10% (2/1,977), respectively (Table 4). Of the 1,977 clinical samples, there were 1,828 tissue samples and 149 whole blood samples. The positivity rates of PHEV, PRV, CSFV, and JEV in tissue samples were 1.37% (25/1,828), 0.38% (7/1,828), 0.88% (16/1,828), and 0.11% (2/1,828), respectively. In the whole blood samples, the positivity rates of PHEV, and CSFV were 4.03% (6/149) and 3.36% (5/149), respectively, while no sample was positive for PRV, and JEV (Table 5).

**Table 4:** The results of clinical samples detected by the developed quadruplex qRT-PCR.

Source	Positive samples				Total
	PHEV (%)	PRV (%)	CSFV (%)	JEV (%)	
Slaughterhouse	12/1,184 (1.01%)	5/1,184 (0.42%)	11/1,184 (0.93%)	0	28/1,184 (2.36%)
Harmless treatment plant	17/388 (4.38%)	2/388 (0.52%)	9/388 (2.32%)	2/388 (0.52%)	30/388 (7.73%)
Pig fram	2/405 (0.49%)	0	1/405 (0.25%)	0	3/405 (0.74%)
Total	31/1,977 (1.57%)	7/1,977 (0.35%)	21/1,977 (1.06%)	2/1,977 (0.10%)	61/1,977 (3.09%)

**Table 5:** The detection results of different types of clinical samples.

Types	Samples	Positive samples			
		PHEV (%)	PRV (%)	CSFV (%)	JEV (%)
Tissue samples	1,828	25 (1.37%)	7 (0.38%)	16 (0.88%)	2 (0.11%)
Whole blood samples	149	6 (4.03%)	0	5 (3.36%)	0
Total	1,977	31 (1.57%)	7 (0.35%)	21 (1.06%)	2 (0.10%)

To validate the feasibility of the quadruplex assay, all the 1,977 clinical samples were also tested with the multiplex qPCR assays reported by Wang et al. (for PHEV) (31) and Wu et al. (for PRV, CSFV, and JEV) (38). The positivity rates of PHEV, PRV, CSFV, and JEV were 1.52% (30/1,977), 0.35% (7/1,977), 0.96% (19/1,977), and 0.10% (2/1,977), respectively (Table 6). The coincidence rates of these assays were 99.95, 100, 99.90, and 100% (Table 6), respectively.

**Table 6:** Agreements between the quadruplex assay and the reference assays.

Method	Positive samples			
	PHEV (%)	PRV (%)	CSFV (%)	JEV (%)
The developed assay	31/1,977 (1.57%)	7/1,977 (0.35%)	21/1,977 (1.06%)	2/1,977 (0.10%)
The reported reference assay	30/1,977 (1.52%)	7/1,977 (0.35%)	19/1,977 (0.96%)	2/1,977 (0.10%)
Agreements	99.95%	100%	99.90%	100%

In addition, the clinical samples were also tested by the qPCR (42), qRT-PCR (43), and RT-PCR (44) in the WOAH’s Terrestrial Manual for PRV, CSFV, and JEV, respectively, and the qRT-PCR in the local standards of Jilin province in China for PHEV, and the detection results of the developed quadruplex qRT-PCR were consistent with the results of the qPCR/qRT-PCR in the WOAH’s standards (data not showed).

## 4. Discussion

PHEV, as one of the earliest discovered porcine coronaviruses, has been prevalent in pig herds worldwide for several decades. Since the infected adult pigs usually show subclinical infections, its damage to pig industry is usually neglected. However, the piglets under 4 weeks of age might be fatal once they are infected, with a fatality rate as high as 100% (4). To date, there is no effective vaccine for this disease, and the piglets can only obtain the specific resistance to PHEV by colostrum antibodies (2, 6, 45). When pigs are infected with PRV, the adult pigs usually develop respiratory symptoms and/or reproductive disorders, while the piglets generally die of central nervous system disease (9). Due to wide vaccination with PRV-gE-gene-deleted vaccine, PR has been successfully eradicated in some countries of North America and Europe, but PR is still a major problem in areas with high pig density in China because of the high prevalence of PRV variants (12–14, 46, 47). Among the several types of pathological changes caused by CSFV, the acute CSF is usually characterized by severe neurological symptoms of depression and anorexia, high fever and various inflammations (48). The vaccination of attenuated vaccine for CSFV and the phase-out policies are generally used to prevent and control CSF (18, 19). However, even though CSF has been steadily controlled in many countries, it is still sporadic or even endemic in some countries (20, 21, 49). For example, there were 285 outbreaks of CSF in 12 provinces in China in 2011 (50). Pigs, as magnified hosts of JEV, usually do not show obvious clinical symptoms when they are infected with JEV, but it has also been reported that JEV may cause fever, anorexia, and reproductive disorders in pigs (26). Nowadays, JE has been steadily controlled through improved vaccination programs, living standards, and sanitary conditions in many countries (28). However, JE is still prevalent in mosquito-infested seasons in some pig herds in China, and one survey showed that the serum-positive rate to JEV is as high as 39.4% during 2006–2012 (51). Therefore, the prevalence of these four pathogens remains serious in some countries around the world, and great efforts are still needed to effectively prevent and control these diseases.

Pigs infected with PHEV, PRV, CSFV, and/or JEV might cause neurological symptoms and encephalitis, showing similar manifestations and pathological damages, so it is difficult to correctly distinguish and diagnose these diseases only depending on the symptoms and damages. Furthermore, pigs are usually co-infected with two or more pathogens in the field (38, 52–54), which increases the difficulty for detecting and diagnosing these diseases. Therefore, it is necessary to establish a reliable detection method for accurate differentiation of these viruses to diagnose these diseases. Traditionally, virus isolation and conventional PCR are usually used to identify and detect viruses, but these methods have some disadvantages, such as laborious, time-consuming, and complex operation, easily polluted during operational process, and relatively low sensitivity (55). Nowadays, real-time quantitative PCR (qPCR) has become a popular detection method in the laboratories, since it shows high sensitivity, specificity, convenient operation, and high through-put, and it operates in a closed environment and is less prone to be polluted (30). The multiplex qPCR has been widely used in the veterinary laboratories, since besides the above advantages of the qPCR, it can specifically, sensitively and accurately detect and distinguish multiple viruses in one reaction at the same time, which saves more time, effort, and expenses (30). As for PHEV, CSFV, PRV, and JEV, some single qPCR/qRT-PCR for detection of these four viruses have been reported, respectively (31–37). The multiplex qRT-PCR to simultaneously detect two to three pathogens of PHEV, PRV, CSFV, and/or JEV have also been developed (38–40, 56). However, no multiplex qRT-PCR has been reported to detect and distinguish PHEV, PRV, CSFV, and JEV until now. In our study, taking the conserved regions of the N gene of PHEV, gB gene of PRV, 5'UTR of CSFV, and NS1 gene of JEV as the target for amplification, four pairs of specific primers and corresponding probes were designed, and a quadruplex qRT-PCR was established for simultaneous detection and differentiation of PHEV, PRV, CSFV, and JEV. The LOD was 1.5 × 10^1^ copies/μL (final reaction concentration), the CVs of the intra- and the inter-assay were both less than 1.84%, the assay did not generate any positive fluorescent signal from twelve other porcine viruses except for PHEV, PRV, CSFV, and JEV, indicating high sensitivity, specificity, and repeatability of this method. This method achieves the goal of using a set of reaction systems to simultaneously detect four viruses in a single test tube at the same time, and indicates highly sensitive, repeatable, and specific. The assay is suitable for application in different laboratories to detect PHEV, PRV, CSFV, and JEV in the clinical samples.

The 1,977 clinical samples collected from Guangxi province were tested by the quadruplex qRT-PCR. The positivity rates of PHEV, PRV, CSFV, and JEV were 1.57, 0.35, 1.06, and 0.10%, respectively, with the coincidence rates of more than 99.90% with the qPCR assays reported by Wang et al. (31) and Wu et al. (38). The results indicated that PHEV, PRV, CSFV, and JEV were still sporadic or popular in Guangxi province, China. Overall, PHEV, PRV, CSFV, and JEV are still popular in many countries around the world, and cause different degrees of damages to the pig industry (5, 6, 13, 14, 20, 21, 28, 29, 57). In China, some provinces have recently reported the prevalence of PHEV, PRV, CSFV, and JEV (41, 47, 51, 58–65). The nasal cavity or throat swabs from 1- to 3-week-old piglets in Jilin province in 2010 showed a positivity rate of 44.23% (207/468) for PHEV (58). The PHEV genome sequences accounted for 75.28% of the identified five coronaviruses of PEDV, PDCoV, PHEV, PRCV, and TGEV in the nasal swabs and serum samples from slaughterhouses in 13 provinces of China in 2017 (59). The samples from different pig farms in 27 provinces in China during 2012 and 2017 showed the average positivity rate of wide-type PRV of 8.27% (1,345/16,256) (46). The serum samples from Henan province from 2019 to 2021 showed the overall positivity rate of PRV-gE antibody of 20.33% (7,276/35,796) (47). The samples from Heilongjiang province showed a serum positivity rate of PRV-gE antibody of 16.3%, and a tissue positivity rate of PRV of 17.8% (51). The samples from Yunan (during 2017–2021), and Hunan (during 2020–2021) provinces showed 31.37% (6,324/20,158) and 10.33% (43/416), and 9.11% (1,840/20,192) and 2.33% (470/20,192) seropositivity rate of PRV-gE antibody, and tissue positivity rate of PRV-gE nucleic acid, respectively (60, 61). The clinical samples collected in Guangxi (during 2018 to 2021), Yunnan (during 2015 to 2021), and Hunan (during 2019 to 2021) provinces of China showed the positivity rate of 9.36% (107/1,143), 3.37% (11/326), and 12.29% (50/407) for CSFV, respectively (41, 62, 63). A total of 22,343 serum samples from 18 provinces of China during 2006 to 2012 showed a JEV-positivity rate of 39.4% (8,807/22,343), with 23.0 to 59.2% in different years (51). The tissue samples from Sichuan (during 2020 to 2021), and Hunan (during 2019 to 2021) provinces showed a positivity rate of 6.49% (12/185), and 10.99% (42/382) for JEV, respectively (64, 65). These reports demonstrated that even though the CSFV, PRV, and/or JEV vaccines have been widely used, these viruses are still prevalent in some pig herds in different provinces of China, indicating that the vaccines are unable to fully protect against the emergent viral strains. Therefore, the established assay in this study can provide a rapid, convenient, and accurate method for detection of these viruses, thus providing a basis for subsequent prevention and control measures.

It is noteworthy that six coronaviruses, PEDV, PDCoV, PHEV, PRCV, TGEV, and swine acute diarrhea syndrome coronavirus (SADS-CoV), have been proved to be the etiological agents to pig. Since the adult pigs infected with PHEV usually show subclinical infections, it is easy to neglect its harm to the pig industry. In this study, the positivity rate of PHEV in Guangxi province was 1.57% (31/1,977), indicating that PHEV is an important pathogen in pig herds at present. Since PHEV might be fatal to the under 4-week-old piglets (2–4, 66), and no effective drug and specific vaccine is available for use now, it should pay more attention to this pathogen, and take effective action to prevent and control this disease.

## 5. Conclusion

PHEV, PRV, CSFV, and JEV cause neurological symptoms in infected pigs, and have caused huge economic losses all over the world. Our study established a convenient, sensitive, specific, and reproducible quadruplex qRT-PCR that can be used to detect PHEV, PRV, CSFV, and JEV in one reaction at the same time. This method can simultaneously detect and differentiate PHEV, PRV, CSFV, and JEV. Furthermore, PHEV, PRV, CSFV, and JEV are still prevalent in some pig herds in Guangxi province of China.

## Data availability statement

The raw data supporting the conclusions of this article will be made available by the authors, without undue reservation.

## Ethics statement

The animal studies were approved by Guangxi Center for Animal Disease Control and Prevention. The studies were conducted in accordance with the local legislation and institutional requirements. Written informed consent was obtained from the owners for the participation of their animals in this study.

## Author contributions

XH: Investigation, Methodology, Writing – original draft. SF: Investigation, Methodology, Supervision, Writing – original draft. KS: Funding acquisition, Supervision, Writing – review & editing, Project administration, Resources. YS: Investigation, Methodology, Writing – original draft. YY: Software, Validation, Data curation, Writing – original draft. FL: Software, Supervision, Validation, Data curation, Writing – original draft. XW: Methodology, Software, Validation, Data curation, Writing – original draft. ZL: Project administration, Resources, Writing – review & editing.

## Footnotes

^1^https://www.ncbi.nlm.nih.gov/nucleotide/

^2^https://www.woah.org/fileadmin/Home/eng/Health_standards/tahm/3.01.02_AUJESZKYS.pdf

^3^https://www.woah.org/fileadmin/Home/eng/Health_standards/tahm/3.08.03_CSF.pdf

^4^https://www.woah.org/fileadmin/Home/eng/Health_standards/tahm/3.01.10_JEV.pdf

^5^https://std.samr.gov.cn/db/search/stdDBDetailed?id=D7B6669FC4B09C41E05397BE0A0AD139
