# Supplementary material for: Development of a quadruplex real-time quantitative RT-PCR for detection and differentiation of PHEV, PRV, CSFV, and JEV
Source: Front Vet Sci. 2023 Oct 31;10:1276505. doi: 10.3389/fvets.2023.1276505 (PMC10643766; doi:10.3389/fvets.2023.1276505)
Supplement: Supplementary file 1 [file Data_Sheet_1.docx]

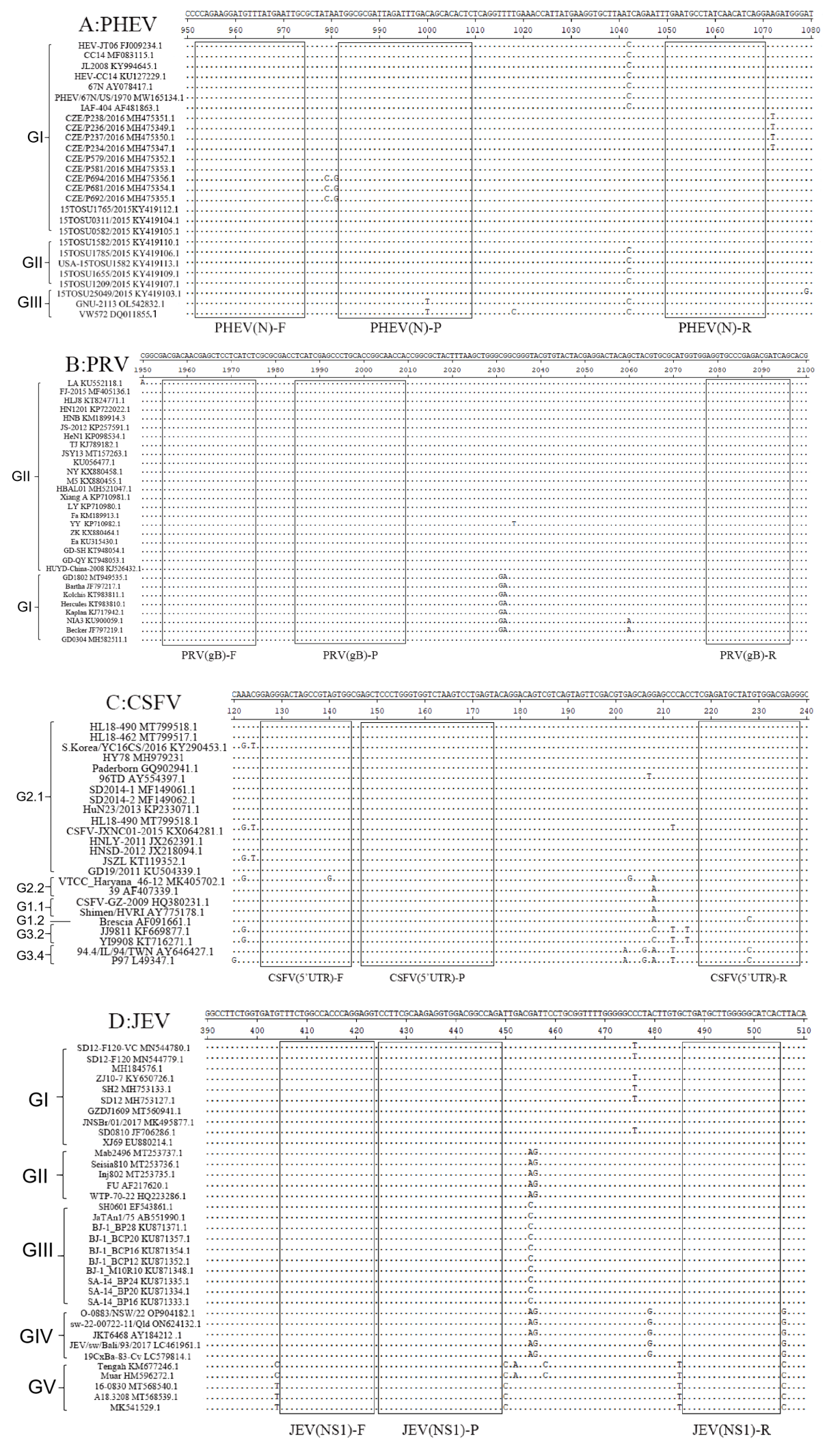


**Figure S1.** Primer and probe location of the quadruplex qPCR. The nucleotide sequence alignments of the partial N gene of PHEV (A), gB gene of PRV (B), 5' UTR of CSFV (C), and NS1 gene of JEV (D) show the locations of primers and probes. F, P, and R indicate the forward primer, TaqMan probe, and reverse primer, respectively.

**Table S1.** The sequences of targeting fragments for construction of plasmids for PHEV, PRV, CSFV, and JEV

| **Virus** | **Reference Strain** | **Sequence** | **Product/bp** |
| --- | --- | --- | --- |
| PHEV | HEV-JT06  (FJ009234.1) | CCAGAAGGATGTTTATGAATTGCGCTATAATGGCGCGATTAGATTTGACAGCACACTCTCAGGTTTTGAAACCATTATGAAGGTGCTTAACCAGAATTTGAATGCCTATCAACATCAGG | 119 |
| PRV | LA  (KU552118.1) | ACGACAACGAGCTCCTCATCTCGCGCGACCTCATCGAGCCCTGCACCGGCAACCACCGGCGCTACTTTAAGCTGGGCGGCGGGTACGTGTACTACGAGGACTACAGCTACGTGCGCATGGTGGAGGTGCCCGAGACGATCAG | 142 |
| CSFV | HL18-490  (MT799518.1) | GAGGGACTAGCCGTAGTGGCGAGCTCCCTGGGTGGTCTAAGTCCTGAGTACAGGACAGTCGTCAGTAGTTCGACGTGAGCAGGAGCCCACCTCGAGATGCTATGTGGACGAGG | 113 |
| JEV | SD12-F120-VC  (MN544780.1) | TTTCTGGCCACCCAGGAGGTCCTTCGCAAGAGGTGGACGGCCAGATTGACGATTCCTGCGGTTTTGGGGGCTCTACTTGTGCTGATGCTTGGGGGCATCAC | 101 |
